# Coordination as Management Response to the Spread of a Global Plant Disease: A Case Study in a Major Philippine Banana Production Area

**DOI:** 10.3389/fpls.2019.01048

**Published:** 2019-08-30

**Authors:** Marilou O. Montiflor, Sietze Vellema, Larry N. Digal

**Affiliations:** ^1^Knowledge, Technology and Innovation Group, Wageningen University & Research, Wagenignen, Netherlands; ^2^School of Management, University of the Philippines Mindanao, Davao, Philippines

**Keywords:** fusarium wilt, Southeast Asia, cross-sector partnerships, plant disease management, Foc TR4

## Abstract

An integrative management approach to the spread and emergence of global plant diseases, such as the soil-borne fungus Fusarium oxysporum f. sp. cubense tropical race 4 (Foc TR4), entails a combination of technical measures and the responsiveness and awareness of area-specific constellations supporting conditions conducive to interactions and coordination among organizations and actors with different resources and diverse interests. Responses to banana diseases are mostly studied through technical and epidemiological lenses and reflect a bias to the export industry. Some authors, however, indicate that cross-sector collaboration is crucial in responding to a disease outbreak. Earlier studies on the outbreak of diseases and natural disasters suggest that shared cognition and effective partnerships increased the success rate of response. Hence, it is important not to focus exclusively on the impacts of a pathogen at farm or field level and to shift attention to how tasks and knowledge are coordinated and shared. This paper aims to detect whether and how the emergence of Foc TR4 is a driver of coordination. The case study focuses on the interactions between a variety of banana producers and among a range of public and private actors in southern Philippines. The analysis identifies distinct forms of coordination emerging in the context of three organizational fields responding to Foc TR4, which underlie shared capacity to handle and understand the spread of a global plant disease. The research is based on qualitative key informant interviews and document analysis and on observations of instructive events in 2014–2017. Analysis of the composition and actions developed in three organizational fields leads to distinguishing three theory-driven forms of coordination: rule-based, cognition-based, and skill-based. The combination of these three forms constitutes the possibility of a collaborative community, which conditions the implementation of an integrative management approach to mitigate Foc TR4.

## Introduction

The global emergence and spread of Fusarium wilt, caused by *Fusarium oxysporum* f. sp. *cubense* tropical race 4 (Foc TR4), threaten both local food securities related to the cultivation and availability of a wide range of banana varieties and the mainly large-scale production of bananas for international trade (Ordoñez et al., 2015; Ploetz et al., 2015; Mostert et al., 2017). An earlier spread of the Foc devastated commercial production of the “Gros Michel” variety in Central and Latin America in the 20^th^ century. The Cavendish sub-group was resistant to this earlier strain of the Foc TR4. However, the current strain of the pathogen that emerged in Southeast Asia in the 1990s also endangers the Cavendish subgroup, and thus the commercial interests of major banana companies (Ploetz, 2015). Foc TR4 infects a range of banana varieties grown under both tropical and subtropical conditions (Buddenhagen, 2009). The pathogen has spread in different banana production systems in Asia, Africa, and the Middle East (Ploetz, 1994; Jeger et al., 1996; Magnaye, 2001; Singh, 2008; Buddenhagen, 2009; García-Bastidas et al., 2013; Ordoñez et al., 2015; Ploetz, 2015). The TR4 is a soil-borne pathogen, microscopic, lacks visible symptoms on suckers and fruits (Brunschot, 2006), displays observable symptoms in the plant during its advanced stage (Ploetz, 1994; Buddenhagen, 2009), and cannot be controlled by a fungicide (Dita et al., 2010). Methods of contamination are not exhaustive but some observations indicated that it may be transferred through infected suckers, soil, surface water, and farm implements (Ploetz, 2015). Zheng et al. (2018) attribute this to the biological complexity of the soil-borne disease, and Dita et al. (2018) emphasize the complex epidemiological dynamics of the fungus that can survive in the soil for a long time (Li et al., 2013). Quarantine and good hygiene practices are means of control currently implemented in affected areas, which is according to Warman and Aitken (2018) especially important in the context of the absence of commercially viable cultivars resistant to Foc TR4 and lack of effective fungicide. Numerous efforts by private and public actors in the commercial banana export sector to contain the spread of the global disease had only a limited effect (Zheng et al., 2018). The responses of other, small-scale banana growers are largely unknown: they can leave infected plants in the field or convert their farms to other crops, and consequently, the pathogen remains in the soil. Hence, from a technical perspective, management of the disease has proven to be difficult, and no cures are readily available.

This limited capacity to control the disease and to contain the spread of Foc TR4 importantly conditions the implementation of an integrative disease management approach (Dita et al., 2018). Research and management approaches addressing the spread of TR4 have adopted a strong focus on plant and field levels. However, some authors in the plant sciences point out that there is a need to go beyond merely technical measures at field level. Ordoñez et al. (2015) and Pocasangre et al. (2011) mention action plans such as awareness campaigns, quarantine measures, the mobilization of joint investments in research, and knowledge exchange in workshops. Accordingly, the feasibility and effectiveness of an integrative management approach also depend on the responsiveness and awareness of area-specific constellations of diverse actors, such as small-scale and large banana producers, industry associations, local government bodies and plant protection organizations, researchers, and extension workers. Therefore, our research complements technical management responses with strategies that emphasize procedures for decision-making and sharing responsibilities and for improving preparedness, horizon scanning, and contingency plans (Ward, 2016). Ward (2016) argues that it is important not to focus exclusively on the impacts of a pathogen at farm or field level and to shift attention to how tasks and knowledge are coordinated and shared at area levels. Consequently, plant disease management strategies are affected by contextual epidemiological dynamics and imply coordinated efforts between different actors embedded in spatially bounded areas with diverse production systems.

The spread of Foc TR4 into areas with multiple banana production systems, e.g., including backyard farming, and production for local markets and for export complicates finding a coordinated response to its spread as well as searching possible control measures. Moreover, in the case of Foc TR4, uncertainties regarding containment of the spread or management of the disease hamper coordinated and integrative plant disease management. The epidemiology and management of other plant diseases, in particular the Banana Xanthomonas Wilt (BXW) that has spread throughout most of East Africa, are equally challenging, and research confirms the importance of understanding emerging forms of coordination for area-based responses to plant diseases (Shimwela et al., 2016; Shimwela et al., 2017). Similarly, the analysis by McAllister et al. (2015) of the Australian response to the outbreak of the fungal pathogen black sigatoka (*Mycosphaerella fijiensis*) demonstrates that success of response was credited to effective partnerships, which mobilized local action. This is in line with the findings of Macleod et al. (2016), who report on consultations among plant protection authorities and identified cooperation among stakeholders and along supply chains as a key mechanism to strengthen weak links, develop spatially explicit integrated decision-making, and reduce plant health risks, especially those moved in trade and in a highly connected world. This is particularly relevant because Foc TR4 ripples through the system and crosses spatial, organizational, and administrative boundaries, which complicates coordinated endeavors and the emergence of novel trajectories supporting resilience to global plant diseases in production areas or communities.

Our research builds on the work of De Vos et al. (2016) and aims to detect whether and how the emergence of Foc TR4 is a driver of new forms of coordination. The Foc TR4 not only connects the pathogen and their hosts (bananas), but also affects other components of the system and their mutual relationships. This mix of complexities requires a typical multidisciplinary approach that this paper endeavors to elaborate. Understanding the coupling of human actions and the biology and epidemiology of pathogens (Berbés-Blázquez et al., 2014) benefits from adopting a landscape or area perspective (Meentemeyer et al., 2012). Accordingly, we adopt an area lens to assess whether the recurrence of Foc TR4 reshapes the interactions between public and private stakeholders and may eventually generate conditions conducive for coordination and collaboration.

This paper presents a qualitative case study, with well-defined spatial and temporal boundaries, of emerging forms of coordination in distinct organizational fields in a major banana production area in southern Philippines severely affected by TR4 (Bureau of Plant Industry, 2012; Molina et al., 2016). The paper qualitatively maps responses to and interactions around pathogen incursions shaped in both formal organizational structures and informal collaborations (McAllister et al., 2015). The case study concentrates on the period in which the manifestation and dramatic effects of TR4 were widely discussed by various stakeholders in the industry: 2014–2017. It traces emerging forms of coordination in different organizational fields observed in the banana sector in the southern part of Mindanao, the most southern island in the Philippines. For this paper, we define coordination as the self-organization and mobilization of responses aligned with those of other actors (Comfort, 2007). The notion of organizational field origins in the work of DiMaggio and Powell (1983) and refers to sets of organizations that, in the aggregate, constitute a recognized area of institutional life that gives direction to problem-solving strategies and sense-making of unanticipated risks and uncertainties. The unknowns related to Foc TR4 as well as the limitations of past experiences to respond to the disease are addressed in the evolving interactions in an organizational field. For this study, we drew empirical boundaries around inductively identified organizational fields anchored in (i) industry alliances and formalized partnerships, (ii) *ad hoc* organizational fields facilitating knowledge exchange and shaping public discourse, and (iii) less salient professional and cross-sector collaborations in which experts share coping strategies for navigating risks and uncertainties related to Foc TR4. We describe the actors and interactions in each organizational field to detect specific emerging forms of coordinated efforts responding to the global pant disease threatening a range of banana producers assembled in a single geographical area.

The paper continues as follows. The next section presents the research area and describes the qualitative and case study methods used for data collection and analysis. The section *Results* identifies and describes three organizational fields that were selected after preliminary research as empirical domains for tracing emerging processes of coordination. In each field, different stakeholders co-create responses to the spread and emergence of Foc TR4. The descriptions of these fields identify the involved actor groups and their connections, the sequential actions and events, and the form of coordination visible. The discussion section 4 matches the empirically identified forms of coordination with theoretically relevant building blocks. To qualify each emerging forms of coordination, we made the connection to theoretical accounts from management, organization, and public management science. The conclusion it explores whether these forms of coordination may configure a collaborative community, which potentially makes an area capable of developing an integrative management approach to Foc TR4 specifically, and other plant diseases more generally.

## Materials and Methods

### Research Area

The case study focused on interactions between a variety of banana producers and among a range of public and private actors in the leading production area of export banana in the Philippines: southern Mindanao. Since the 1960s, the island of Mindanao in southern Philippines emerged as the country’s main producer of bananas, contributing to 82.8% of the country’s total volume in 2015. Mindanao produced almost all of the country’s Cavendish-AAA (*Musa acuminata*), 84.8% of the Lakatan-AA (*Musa acuminata*), and 55.6% of the Saba-ABB (*Musa acuminata* x *Musa balbisiana*) (Philippine Statistics Authority, 2017). The Davao Region produced half of the Cavendish variety in 2015 (Philippine Statistics Authority, 2017).

Plant diseases have been a major problem for both the export industry and producers serving domestic markets. Besides the recurrence of Foc TR4, the following diseases were reported: Moko, black sigatoka, (Stover, 1986), nematodes (Davide, 1988; Herradura et al., 2012; Aggangan et al., 2013), and bunchy top (Kohnen et al., 2013; Molina et al., 2009). The Foc TR4 had been reported in Mindanao since 2009 and affected a range of banana production systems located in the area. The production systems were connected as adjacent farms or *via* the movement of people, banana fruits or leaves, vehicles, and farm tools and machinery (Ploetz, 2006). In 2015, Foc TR4 affected an area of around 15,500 hectares of banana production in Davao Region, which accounted for 32% of the total Cavendish production area (**Table 1**).

**Table 1:** Estimated areas in Davao Region affected by TR4 in 2015 (in hectares).

	Banana (all varieties)	Cavendish	Area affected with TR4	Percentage of Cavendish area affected with TR4
Philippines	443,369.91	85,808.90	Not available	Not available
Davao Region	88,274.80	48,050.00	15,507.53	32.27
Davao del Norte	036,368.00	28,972.00	13,743.00	47.44
Davao del Sur	15,413.00	3,642.00	436.00	11.97
Davao Oriental	10,528.80	156.00	36.00	23.08
Compostela Valley	19,131.00	11,934.00	1,082.78	9.07
Davao City	6,834.00	3,346.00	209.75	6.27

Sources: Department of Agriculture; Philippine Statistics Authority.

This multi-actor landscape made the area interesting for a study of coordination: a variety of stakeholders tried to find ways to respond to the spread of Foc TR4 (Pocasangre et al., 2011) and developed distinct strategies to control plant diseases (de la Cruz and Jansen, 2018). Disease management in banana plantations has been one of the controversial issues, specifically concentrating on the unintended effects of aerial spraying on the health of workers and communities (Panganiban et al., 2004). Large banana plantations have their own research departments while smallholder farmers receive information from government line agencies through the local government. Aside from farmers and corporations, other actors also influenced and co-created trajectories for managing plant diseases (de los Reyes and Pelupessy, 2009; Dalayon, 2013). These included commercial input and service providers and strategic alliances of academe, government, and financial institutions. In addition, the presence of major city in the banana production area connected consumers directly to disease management practices in banana farms, which paved the way for strict regulation of pesticide use and promoted organic production (Davao City ordinance No. 0384-10, 2010).

In addition to distinct perspectives on disease management, the area featured a diverse landscape of banana producers with different landholdings and sometimes opposed interests and perspectives. (De Leon and Escobido, 2004; Borras and Franco, 2005; Digal, 2007; Lockie et al., 2015). The export banana production on large and medium plantations expanded since the 1960s. The industry became influential toward policy and developed infrastructure for international trade, such as roads and ports. Large plantations were either family-owned or managed by multi-national corporations. These corporations were mostly members of the Pilipino Banana Growers and Exporters Association (PBGEA). The export-oriented industry produced bananas on managed plantations, used contractual arrangements with cooperatives and individual growers, or sourced directly from smallholder farmers (Digal, 2007; de la Cruz and Jansen, 2018). This was partly a response of the industry to land reform program implemented by the Philippine government. Coordination and collaboration between the export industry and agricultural producers supplying other markets were limited.

### Case Study of Emerging Forms of Coordination

For investigating the possible emergence of coordination, the study adopts an area-perspective approach. The case study method was appropriate for documenting specific events and tracing unfolding coordination processes situated in a spatially bounded landscape affected by the spread of a global plant disease, Foc TR4. The case study (Yin, 1994) advances a contextual understanding of the degree to which actors with different interests, worldviews, and management styles agreed to coordinate their responses to Foc TR4. The case study involved identifying organizational fields in which distinct groups of actors interact and exchange information and knowledge. In addition, it entailed using process tracing as a method to document procedures that plausibly generate different forms of coordination in real-life situations marked by multiple interaction effects, and where it was difficult to explain coordination as the outcome of a limited number of independent variables (George and Bennett, 2005). The case study revealed temporarily and spatially unique coordination processes, which, as a first step, were inductively identified and subsequently elaborated by matching empirical findings with theory (Flyvbjerg, 2006). Process tracing generated a range of observations and identified the sequences of events, which exposed processes leading to the outcome of our interest: coordination.

### Data Collection

Data collection took place in the years 2014–2017 and involved (i) the study of formalized collaboration agreements, (ii) the observation of *ad hoc* interactions during public events, (iii) the documentation of career histories and professional affiliations of key players, and (iv) document analysis of information shared in media and trainings.

The first step was to collect data on formalized arrangements assembling key private sector actors associated with the export banana industry, in particular the Pilipino Banana Growers and Exporters Association (PBGEA, representing larger corporations) and Mindanao Banana Farmers & Exporters Association (MBFEA; a platform assembling small and medium Cavendish farmers and exporters). This was based on one structured interview, with a representative of the association, and semi-structured and unstructured interviews with members during public events.

Second step was to collect data during public events, which were considered to be *ad hoc* and temporary organizational fields bringing together multiple stakeholders from private and public sectors, non-governmental organizations (NGOs) working with smallholder farmers, and representatives of organizations and cooperatives formed by agrarian reform beneficiaries. From 2014 to 2016, there were four banana events observed which focused on Foc TR4 (**Table 2**). These events provided venues for the examinations of spontaneous (unrehearsed) interactions among the actors. Participation in these events allowed observation of the ways in which stakeholders discussed coping strategies and a menu of solutions to address the spread of Foc TR4. Thirty six unstructured interviews consisted of conversations with industry actors during field visits, workshops, symposia, and conferences. Unstructured interviews with randomly chosen industry actors lasted between 15 and 30 min. Their responses became entries to conversational journals, which contained notes and narratives to provide context

**Table 2:** Summary of methods of data collection.

Methods	#	Source	Focus
Key informant interviews	21	-3 university employees/researchers -10 local/national government employees -3 NGO employees -2 private sector representatives -3 farmers	-Involvement with the banana industry -Activities related to banana and Foc TR4 -Perceptions/opinions about banana-related issues
Ad hoc unstructured interviews	36	-14 local/national government employees -15 farmers -5 private sector representatives -1 NGO employee -1 university employee	-Familiarity with the Foc TR4 problem -Interactions with other industry players -Perceptions/opinions about banana-related issues
Observations	4	-International Stakeholder Workshop (February 2014, Davao City, Philippines) -International Banana Symposium (November 2014, Davao City; estimated 568 participants) -International Banana Congress (April 2016, Miami, Florida, United States of America; estimated 600 participants) -8^th^ International Conference on Agribusiness Economics and Management (October 2016, Davao City, Philippines; 218 participants)	-Interactions with other industry players -Studies about Foc TR4 -Solutions and mitigating actions -Perceptions/opinions about banana-related issues
Documentation of media coverage	128	-Online newspapers -Websites	-Solutions and mitigating actions -Identification of actors (specific names, sector, or organizations) -Observation of signals of coordination and blaming

The fourth step consisted of interviews with a purposefully selected sample of key actors from the private and public sector active in 21 in-depth key-informant interviews provided insights into their educational and career histories, their linkages and interactions with professionals in public and private sectors, and their approaches to the spread and management of Foc TR4. Key informant and unstructured interviews were recorded and transcribed with consent from the respondents. Answers of those who refused to be recorded were noted and encoded. The languages used in the interviews were English, Cebuano, Filipino, and Ilonggo/Hiligaynon. The quotes were translated in English in this paper. To protect the anonymity of the research participants, their names were withheld, identified only by their sector, location, and date of interview.

The final step involved reviewing 128 media reports and training material, which served as a source for documenting how Foc TR4 was discussed in the media and whose perspectives and views were represented.

### Data Analysis

The interviews, observations, and document analysis resulted in indications of how actors with different interests approached coordination and tensions around TR4 spread. Data analysis involved three steps (**Table 3**): (i) qualitative mapping of the actors and their interactions, (ii) constructing time paths of events and activities, and (iii) identifying the variation of responses to Foc TR4. Firstly, we determined what types of actors were connected in the three identified organizational fields. The key informant and informal interviews were transcribed, then manually scanned to find themes, compared it with the responses of other respondents on the same topic, and analyzed the answers. There were observed recurring names mentioned during the interviews, media releases, and public events. Based on media reports, structured and unstructured key informant interviews, the career histories, and visualization of the professional networks, the recurring names of actors were listed, and mutual linkages were detected. It was observed that these individuals moved in the same circles because they belong to the same professional group, shared the same alumni network, worked together in previous projects or company, or served as keynote speakers in a conference. Secondly, chronological and thematic clustering of events was mentioned in the interviews, outlining the activities and discovering indications of coordination in these documented and observed processes. In addition, during this second step, the observed dialogues at public activities and in media releases were analyzed to determine the approaches of banana companies, government line agencies, and local government units (LGUs) in detecting and mitigating Foc TR4. Finally, a selection of instructive events were investigated in more detail to discover the specific types of responses to Foc TR4 emerging in each of the organizational fields and to establish who formulated these responses.

**Table 3:** Qualitative data analysis.

Step	Focus of the analysis
Step 1. Examine who are the actors in the organizational field	To discover what groups or individuals are in the organizational field
Step 2. Observe and examine the activities of the organizational field	To map the actions developed in the organizational fields and establish the sequence of events
Step 3. Derive patterns of coordinated activities and typify the response	To distinguish emerging forms of coordination

Next, the qualitative case study analysis followed the process proposed by Ruona (2005, p. 236): (1) sensing themes, (2) constant comparison, (3) recursiveness, (4) inductive and deductive thinking, and (5) theory-informed interpretation to generate meaning and typify emerging forms of coordination. The properties of the empirically detected forms of coordination were elaborated by making a connection to a selection of theoretical explanations of why and how different actors agreed to act in a coordinated manner. Building on Ruona, (2005) process of analyzing qualitative data, we identified three empirical manifestations of organizational fields: the industry-based and formalized partnerships, temporary and *ad hoc* multi-stakeholder settings, and professional associations and networks. The study first focused on formal industry associations with an established history of coordination and advocacy in the banana industry, mainly supported by resourceful and powerful firms working in the export sector. The emergence of Foc TR4, however, induced a series of public events accommodating interactions between stakeholders that did not connect before and now used multi-stakeholder platforms to express their concerns and to agree or disagree on the diagnosis of the origins, implications, and possible management of Foc TR4. The documentation of career histories of key informants in the private and public sectors indicated less obvious interactions taking place, which seemed to be related to a distinct organizational field. These three organizational fields were used to structure the result section and to derive patterns of coordinated activities.

## Results

This section distinguishes three organizational fields in which multiple actors interact to come to grips with the unknowns and uncertainties related to Foc TR4 and to explore and evaluate open-ended solutions for an unprecedented plant disease problem. The three organizational fields are instructive for detecting emerging forms of coordination depicted in each section. The descriptive account of who interacts, how and in what ways, set the stage for matching observed processes and sequences of events with theoretical accounts of coordination in the next section.

### Organizational Field 1: Industry-Based and Formalized Partnerships

Two prominent industry-based and formalized partnerships in the Philippine banana industry were the Pilipino Banana Growers and Exporters Association (PBGEA) and Mindanao Banana Farmers and Exporters Association (MBFEA) Inc. The associations coordinated activities among its corporate members. PBGEA was composed of around 31 large multinational and family-owned corporations (Tourism Promotions Board Philippines, 2017) while MBFEA was composed of around 23 small and medium banana growers, exporters, and federations of cooperatives (Mindanao Banana Farmers & Exporters Association Inc, 2017). PBGEA and MBFEA represented companies predominantly producing and trading Cavendish bananas for export. Both associations have Technical Committees, which focus on Research and Development and on regulatory aspects of managing diseases in the banana industry.

Prior to the emergence of Foc TR4, the associations’ coordinated efforts existed for the management of other diseases such as Black Sigatoka which can be controlled, by fungicide treatment (while TR4 cannot). Additionally, the PBGEA and MBFEA successfully lobbied and advocated for issues beneficial for their corporate members, such as repealing restrictions on the limit of banana hectares, banning aerial spray, and installing stricter policies on agricultural venture agreements. A clear example of joint advocacy is how the PBGEA asserted the illegality of the Davao City Ordinance No. 0309-07: *Banning Aerial Spraying as an Agricultural Practice in All Agricultural Activities by All Agricultural Entities*. This resulted in litigations and dialogues with the private, government, and non-government organizations (NGOs). The local NGOs were supportive of the ordinance and managed a campaign to implement the ordinance and ban aerial spraying. PBGEA, on the other hand, appeared in legislative hearings to push their own agenda. Eventually, in 2016, the Supreme Court ruled Davao City Ordinance No. 0309-07 as unconstitutional (Perez, 2016), and banana companies in Davao City were allowed to engage in aerial spraying. Decisions concerning an “industry-sanctioned” solution needed the agreement of not only the technical officers but all members of PBGEA had to consent. Decision-making within the associations was bounded by rules and based on reaching consensus. When a collective approval was not possible, interested individual companies were approached for collaboration. Since the members were business competitors, some were more cautious when it comes to working with non-members on banana-related issues. One member of PBGEA explained that collaborative projects were not easily supported by PBGEA. This member said to be after the collective interest and look for what is good for the group, but other members may be less open to joint endeavors (Interview with Private Sector, Davao City, 04 November 2016). Any decision regarding collaborative efforts had to pass the bureaucratic procedures installed in the associations.

Foc TR4 has been on the agenda of the industry-based associations, but concerted action was not automatically the outcome of their deliberations. One member of PBGEA explained.

*We nonchalantly talked about Foc TR4. In fact, as early as 2005, someone was already proposing some sort of a Task Force to be organized by the government to monitor the disease and the extent of the infection. That time, he was also proposing that we sort of come up with a war chest to be used to arrest or address this Panama disease. But considering that the industry was facing many problems that time, it did not push through.*

*(Interview with private sector, Davao City, 18 November 2014)*

The technical committees of the associations played an influential role in the decision-making about how to respond to Foc TR4 and with what other actors to align. Members of the associations tried to implement their individual disease management strategies and exchanged experiences during meetings of the association. Existing interdependencies of large plantations and other farms supplying the banana companies also shaped interactions around disease management. For example, both managed/leased farms and those under contract growing utilized aerial spraying as a disease-control method. These managed farms, with an average of more than 300 hectares, tried to control Foc TR4 through quarantine measures, i.e., foot baths at every gate or entrance to the farm, which was also common practice in the larger plantations. Larger companies expressed concerns about adjacent farms, since these have limited technical and financial capacities to address outbreak of Foc TR4, and the pathogen might spread from these farms. Even more so, farms supplying spot markets were considered a threat: these are relatively dispersed compared to farmers under lease and contract-growing arrangements. For area-based management strategies, however, representatives of banana companies recognized that collaboration was crucial for controlling a large track of contiguous farms. This, however, contrasted with the history of working with relatively isolated plantations. During observed meeting of PBGEA, this issue was raised, but no specific actions were prioritized to stimulate area-based coordination.

The industry-based partnership aligned with government line agencies, such as the Department of Agriculture (DA). Politically, the DA had close connections with the banana industry through a series of secretaries coming from the industry, but cross-sector collaborations in the production areas were rare. However, Foc TR4 induced more interactions, in particular around quarantine. The alignment of the industry-based associations and government agencies resulted in a Task Force *Fusarium* initiated by the DA. This task force was a response to the lobbying of the associated Cavendish growers. The Task Force (TF) was composed of government line agencies, banana companies, LGUs, PBGEA, MBFEA, and other stakeholders. One of its functions was to identify stakeholders to collaborate with disease management. The TF made plans for the training of LGUs on the recognition, containment, and handling of Foc TR 4. Furthermore, bureaus and divisions of the DA were mandated to work on specific issues, such as Foc TR4. The regional DA offices coordinated with LGUs, which in turn identified the target beneficiaries of government programs and projects. After the passage of the Local Government Code of 1991 (Republic Act 7160), the agricultural extension was devolved to the LGU’s Provincial/Municipal/City Agriculturist’s Office. The technical committees of the industry-based associations oversaw collaborative activities with external stakeholders in banana plantations, such as universities (Interview with university employee, Davao City, 22 October 2014). In addition, coordinated activities were initiated in villages where plantations were located. There were multi-partite monitoring teams (MMT) involving village leaders, non-government organizations, Fertilizer and Pesticide Authority of the Department of Agriculture, Department of Environment and Natural Resources-Environment Management Board representatives, and heads of banana companies operating in the area. The MMTs were organized to encourage stakeholder vigilance and monitoring of Foc TR4 in the villages concerned. It was also intended to monitor compliance of companies to Philippine environmental laws. Interviews and observations disclose that both the decision-making procedures with the industry-based associations and the alignment of the private sector and public sector suggest a form of coordination building both on bureaucratic procedures to agree on resolutions and joint actions and regulatory interventions meant to monitor Foc TR4 and explore conditions for installing quarantine measures.

### Organizational Field 2: Temporary and *Ad Hoc* Multi-Stakeholder Settings

The Foc TR4 discourses became prominent in the Philippine media and was reported on *ad hoc* knowledge exchanges and interactions such as several banana congresses, workshops, and symposiums where multiple stakeholders discussed the implication of and possible solutions to Foc TR4 (**Table 4**). Some of these exchanges were public while others were by invitation only. We observed interactions in three multi-stakeholder settings in Davao City: International Workshop on Panama Disease in February 2014, International Banana Symposium in November 2014, and the 8^th^ International Conference on Agribusiness Economics and Management (ICAEM) in October 2016. Aside from banana producers, other participants came from government agencies, LGUs, non-government organizations, funding agencies, academe, and input suppliers.

**Table 4:** Examples of *ad hoc* encounters and interactions.

Type of action	Description	Participants	Focus of actions
Research collaborations	Collaborative and interdisciplinary research between private and public sectors	Universities, Inter-government (i.e., Department of Agriculture, Department of Science and Technology), plantations, individual growers, smallholder farmers, research institutes, local government units	Coming up with technical solutions, new varieties, quarantine measures, and experiments
International Banana Symposium/congress	Sharing of banana related issues, ranging from pests, plant, people, and the environment	For example, in November 2014: 25 International and local speakers with 568 delegates	Public debate of veracity of the problem, which solutions to use and who is to blame
Workshops and training	Usually among partners, long-time or new collaborators	Selected people/partners, depending on the type of workshop	Knowledge sharing, information dissemination and identification of possible partners
Meetings	Broader attendance and participation of stakeholders especially if it is industry wide	Selected people/partners, depending on the type of the meeting	More thorough discussion of the issue from different perspectives, not only on the technical side but also on organizations and people involved

Source: Fieldwork, news articles, websites.

While the main purpose of these multi-stakeholder gatherings was to share information and make connections, the public events were also used to make sense of the risk and its effects on a variety of banana producers. Perhaps, more importantly, participants hoped to find solutions to the disease problems destroying their farms, which hampered investigating the many uncertainties and unknowns also mentioned during the presentations and discussions. Stakeholder discussed a variety of mitigating strategies in the *ad hoc* gatherings, such as quarantine measures including setting up of footbaths in designated farm entrance (for humans) and tire baths in roads leading to the plantation (for vehicles). The chemicals vary but many farms used formaldehyde. Other strategies focused on information dissemination, directed mainly to smallholder banana farmers. Although some companies implemented the quarantine measures and the LGU disseminated information, participants in the meetings also recognized that these strategies were short-lived due to budgetary and jurisdiction issues.

The divergent views on how to manage and mitigate Foc TR4 were also a prominent feature of the research inputs to multi-stakeholder meetings. In particular, the use of somaclones, which were claimed to survive Foc TR4 for a certain period, led to debate and controversies, although some major banana companies decided to try this as a solution. When scientists with different perspectives on short- and long-term solutions shared a stage during the 6^th^ International Banana Conference in Miami, Florida, organized by Costa Rica’s National Banana Corporation (CORBANA), and the Association for Research and Integrated Management of Banana and Plantain (ACORBAT), the rebuttals on methods, results, and scientific viability and importance of each other’s contribution were highlighted. Similar tensions between research-based preferences for disease management strategies were observed during the multi-stakeholder meetings in Davao City.

Despite the differences in opinions, the interactions in multi-stakeholder for a created a common concern in Foc TR4 and even stimulated the implementation of joint projects. For example, a project funded by the Department of Science and Technology was about “S&T (Science and Technology) Management Approaches against *Fusarium* (*Fusarium oxysporum* f. sp. *cubense (Foc*) on Cavendish in Mindanao.” It had a budget of ₱34.05 million, with the Department of Agriculture-Bureau of Plant Industry (BPI), University of Southeastern Philippines, and Southern Philippines Agri-Business and Marine and Aquatic School of Technology (SPAMAST) as partners. The program aimed to reduce disease infestation with a focus on adaptability trials of resistant varieties, biological control strategies, and assessment of the distribution of the Foc TR4 in Mindanao. It supported institutions through human resource development, laboratory facilities upgrading, establishment of molecular laboratory, green houses, and other needed facilities and equipment (Valencia, 2015). Another project, funded by the Department of Agriculture, had a budget of ₱102 million and set out to stop the spread and occurrence of Panama disease in Region XI (Valencia, 2015). Since the *ad hoc* forms of collaboration were composed of newly assembled members, team building activities were also included in the project as in the case of the Southern Mindanao Agriculture and Resources Research and Development Consortium (SMARRDEC)’s project on “Science and Technology Management Approaches against Fusarium Wilt (*F. oxysporum* f. sp. *cubense (Foc)* on Cavendish in Mindanao.”

Besides projects, trainings also allowed different stakeholder to interact on an *ad hoc* basis. For example, one training focused on a participatory approach to Foc TR4 management. Participants included farmers, researchers, and extension agents. This training workshop aimed to enhance the capabilities of local researchers, extension agents, and local farmers in providing effective disease management approaches. Other elements of the training were the proper detection of Foc TR4 in the field. Another example was a training on diagnosis and characterization provided to banana companies, universities, research organizations, and government agencies. Resource persons were from Bioversity International, University of the Philippines Los Baños, and Stellenbosch University (South Africa). There were also trainings for diagnosis and knowledge sharing by the Department of Agriculture. The DA shared four interventions to assist banana farmers to mitigate *Fusarium*: cash for work program for eradication, planting of GCTCV 219, crop conversion program, and *Trichoderma* distribution (Maestre, 2015).

These temporary and often *ad hoc* gathering resulted in the exchange of information, but observations also signaled that participants were quite desperate to find solutions. Tensions arose when participants proposed a solution, while others were doubtful about the effectiveness or demanded careful evaluation first. Moreover, the multi-stakeholder meetings were also used to discuss where the disease came from, and consequently who is to blame for the destruction of many banana fields. The multi-stakeholder meetings became avenues for public debate, where turfing, positioning, information-sharing, and blaming mingled. It was noted during the February 2014 workshop that farmers blamed the large companies for denying the spread of Foc TR4 and non-disclosure of the disease’s presence in their plantations. The farmers felt the public information about the disease was late because many bananas were already infected. Moreover, people’s clothes and footwear and farm tools (such as bolos and scythe), which were believed to be carriers of the disease, had been exposed to TR4, and farmers unknowingly contaminated other areas/plants. Meanwhile, an employee during the same workshop blamed the small farmers, working in villages adjacent to their plantation, for the spread and contamination. The employee shared the company’s frustrations that despite adequate efforts to disseminate information (in collaboration with government agencies), farmers and residents were uncooperative. An MBFEA representative, who stated that DA was not supportive to all of banana farmers, which prompted a meeting with some DA Region 11 personnel, other government representatives, and selected banana farmers to clarify the issue. This meeting (details were confidential), which was attended by one of the authors, concluded on the agreement that one farmer leader’s opinion did not represent the majority.

Observations of various temporary and *ad hoc* occurrences of multi-stakeholder encounters not only exposed tensions and different interest and views, but also signaled emerging space for collective sensemaking of the risk for the range of banana producers located in a single area. Despite controversies and tensions, the meetings and related media attention also raised awareness of the unknowns and uncertainties related to management of the disease. The observed interactions also induced recognition of new type of interdependencies underlying any form of collective mitigation of a disease crossing organizational boundaries and affecting large and small banana producers alike.

### Organizational Field 3. Professional Associations and Networks

The interviews and documented career histories of key informants from the private and public sectors indicate that long standing relations were rooted and reproduced within alumni associations and professional networks. These communities were composed of alumni networks from the University of the Philippines (particularly the Los Baños campus), University of Southeastern Philippines, and University of Southern Mindanao—all agricultural universities in the Philippines. The connections among professionals were reproduced by membership of scientific societies such as the Philippine Association of Agriculturists, Crop Science Society of the Philippines, and Philippine Phytopathological Association. Six key informants attended the annual meetings of these associations, which included presentations of new findings and researches. Moreover, the meetings became a venue for sharing ideas, some of which translated into actual projects and collaborations.

The professional associations and networks enabled interactions based on personal ties or social connections. A key informant from the private sector related how he tapped a friend from an international research institute and a local politician to discuss urgent issues and future plans for the banana industry:

*I organized a summit, in which we talked about Fusarium. I tapped my friend who is from the Taiwan Banana Research Institute and I also invited a congressman to talk about the National Banana Research Center. We also talked about the bills regarding pro and anti-aerial spray.*

*(Interview with Private Sector, Davao City, 04 November 2016)*

Another key informant from government indicated that cross-sector interactions in informal professional networks were easier if you were trained at the same university or belonged to the same scientific society:

*I am a member of UP Phytopathological. Of course, if you are from UP (University of the Philippines), even if it’s not plant path, for as long as you are from UP, you will be introduced and access is easier. Especially if you belong to the Philippine Phytopathological Society body.*

*(Interview with National government employee, Davao City, 7 February 2017)*

A university employee explained that mobilizing friendships with other professionals helped to move things forward when formal partnerships were not able to agree on a collective resolution:

*If we want to do things in a corporate farm, they (the corporate farm) cannot decide. They have to go to PBGEA because PBGEA has a governing technical committee. One company allowed us to do research because we have a friend who was the Director of Research and who can allocate a field for trials. In the end, PBGEA only allowed this company to be involved.*

*(Interview with university employee, 22 October 2014, Davao City)*

The interviews revealed that most of the people working in the banana industry, particularly those who were involved in actions addressing Foc TR4, were familiar with each other, both personally and professionally. It was observed that the same people attended similar *ad hoc* actions, research projects, workshops, meetings, and task forces. There were also events where they met as university alumni or as professional group members. The acquaintance made cross-sector collaboration easier; a university employee explained that personal relations with a local government unit enabled access to production areas and regularly organize a forum with farmers and link with partner agencies. Moving through the personal networks was essential for this (Interview with university employee, Davao City, 22 October 2014)

Media reports in newspapers and websites (Philippine News Agency, 2011; Madrazo-Sta. Romana, 2012; Valencia, 2015) expose indications of interactions among alumni, colleagues, and peers within and between organizations opened space to explore contextual and experimental pathways addressing Foc TR4. Industry actors from different companies and government agencies recognized that Foc TR4 did not have a single solution and looked for ways to temper the effect or pragmatically continue banana production (Cayon, 2011; Peña, 2012). This type of response was inclined to look for a combination of disease management practices. Responding to the disease was too complicated, and it was unlikely that a single solution would be available. In unstructured interviews, employees of large-scale banana plantations indicated to adopt quarantine measures at the boundaries of the plantation, work with skilled foremen for detecting diseased plants and eradicate them, support trials with somaclones, recognize that soil management matters (as was suggested by one of the banana cooperatives for agrarian reform beneficiaries), and engage in long-term research projects looking for resistant varieties (Lumawag, 2015). Multiple key informants confirmed that they exchange insights from trial and error procedures with peers working in different sectors. Private sector employees borrowed hands-on measures experimented by peers and were open to evaluate the results together in informal settings.

The interviews indicate how professionals crossed organizational boundaries, both between the public and private sectors, and between large companies and smallholder farmers. In the history of the banana industry in Mindanao, these boundaries used to be less permeable, but the management approaches to Foc TR4 adopted by expert professionals indicate a certain willingness to move outside established silos. Private sector employees reached out to government officials whom they shared a professional affiliation with, in particular plant pathology. In addition, some companies also realized that reaching out to neighboring smallholder banana growers could be part of the response to TR4, which was further complicated by farmers who decided to convert their land to other crops without treating the Foc TR4, and therefore allow the pathogen to stay in the soil.

## Discussion

The previous section identified three organizational fields in which different stakeholders interacted and co-created responses to the spread and emergence of Foc TR4. This section matches the above descriptive accounts with theoretical insights in coordination to further typify the observed emerging forms of coordination. The emerging forms of coordination are labeled as: rule-based, cognition-based, and skill-based.

### Rule-Based Coordination

The organizational field including industry alliances and formal partnerships is rooted in the dominant export sector producing and trading Cavendish. This exclusive organizational field has strong ties to the globalized economy, in which people and products move, through air, water and land. These movements are a factor in spreading plant diseases. These partnerships build on coordinated actions prior to the emergence of Foc TR4, which included lobbying for legislation to lift the ban on aerial spraying. These same adversaries could become allies in other issues, such as searching for mitigating factors and solutions for emerging plant disease threats. However, the issue of aerial spraying mainly relates to the management of Black Sigatoka in large-scale plantations and is different compared to Foc TR4 issue in terms of the coordination requirements. Foc TR4 has the risk of spreading the disease when efforts of players in addressing the issue vary. Resultantly, large firms have good reasons to share their knowledge to those who supply to them under contract as well as to those who supply other buyers (e.g., spot market) as they pose a risk. The presence and the nature of Foc TR4 encouraged the rather cautious and exclusive export-oriented industry players to be open for more opportunities to collaborate.

As leading industry players, this organizational field tried to coordinate among themselves and with regulatory government bodies in order to agree on rules, particularly on quarantine, and to prescribe preferred disease management practices. Regular organizational members/representatives met for meetings or projects, with some information privately and exclusively available to them. Representatives of individual companies behaved and reacted based on their organizations’ directives. The formal partnerships feature bureaucratic interactions and are inclined to set of rules and standards to govern its operations. Formal arrangements such as memoranda of agreement or membership regulations enable coordination (Quero, 2012). The private sector alliance coordinates with the public sector in formulating guidelines and laws and implementing rules and regulation (Leone and Gaillard, 1999). This kind of rule-based coordinated action is structured, procedural, and formal.

### Cognition-Based Coordination

In the *ad hoc* organizational fields of stakeholder meeting during workshop, training, and conference interactions and membership are flexible and cover a wider scope of issues, and the orientation leans toward exchange of information. Since it is an *ad hoc* interaction, its mandates depend on what is collectively agreed by the interim members. This means that protocols, rules, and process are constantly changing, including the functions of participants (individuals or representing their organizations). The more the actors talk and interact with each other, the more effective is their governance (Andersson, 2004). This form of interaction in the form of public meetings and exchanges, also including media coverage, features a degree of inclusiveness. Information, attendance, and memberships are often open and accessible to the public. The *ad hoc* organizational field accommodates interactions that connect actors recognizing the treats affecting many in an area.

This organizational field provides a platform for different stakeholders trying to make sense of the risks and exploring possible solution pathways, and therefore plays a cognitive role in the landscape of actors affected by Foc TR4. Comfort (2007) identifies cognition as a vital ingredient of any concerted action in responding to natural hazards or biological risks affecting a variety of actors in a single area. This is the case with TR4, which has uncertainties and has no universally accepted chemical, biological and cultural cures. Comfort (2007) argues that capacities to communicate, control, and coordinate remain disconnected if cognition is absent. Comfort derives this insight from several studies mostly in the United States of America analyzing responses to natural disasters, such as typhoons or floods, in spatially bounded areas (Comfort et al., 2001; Comfort et al., 2004; Comfort, 2005; Comfort and Kapucu, 2006; Comfort and Haase, 2006). Cognition may start with technical experts working in laboratories of multinational companies or local governments, but normally these experts elevate concerns to their organizations and other stakeholders. This type of expert knowledge has become part of deliberations and interactions taking place in *ad hoc* organizational fields that contribute to the recognition of threats and construct shared or contrasting cognitive understanding of Foc TR4.

### Skill-Based Coordination

This study highlights the invisible and informal form of skill-based coordination as an important resource for navigating the tensions and conflicts around the formal and public responses to the spread of the disease. It identifies professionals that belong to a specific technical profession, i.e., plant pathology, and most often do not hold management positions as key players in this form of skill-based coordination. Bardon et al. (2015) emphasize the relevance of professional association for coordination because peers incorporate shared elements in approaching management problems and interacting with other professionals. This connectivity allows actors to tweak organizational protocols while navigating risks and uncertainties. This form of skill-based coordination entails interactions among alumni, colleagues, and peers within and between organizations (Grindle, 1977; Lawrence, 2004; Mudambi and Swift, 2009; Hatmaker et al., 2011; Noordegraaf, 2011; Levine and Prietula, 2013; Cohen and Malloy, 2014; Bardon et al., 2015). It has the capacity to find and recognize contextual pathways addressing a virulent plant disease, to avoid prescribing single solutions, and to contain the tendency to blame others (Vellema and Jansen, 2018). We observed trust in the form of pursuing the solutions and mitigating actions recommended by colleagues and getting access to people and information, which formed a significant ingredient in informal coordination (Zanini and Migueles, 2013). Cross-sector coordination is importantly realized by a variety of peer groups that are able to cross-organizational boundaries and share informal, symbolic, or task-oriented views associated with a profession.

Following Adler et al. (2008), our findings emphasize this peer community principle as a mechanism underlying hidden forms of coordination that entail careful selection of trusted collaborators. In this way, individuals associated with principles and management views of a specific profession (Mudambi and Swift, 2009; Noordegraaf, 2011) and employed by different organizations mobilize their networks to solve problems and develop collaborative capacity (Kaplan, 2000). These professional associations generate a certain degree of common understanding (Yanow, 2004) and share practices and routines to manage problems (Lawrence, 2004). The skill-based coordinated action is dynamic. It enables actors to cross both organizational boundaries and borders between private and public sectors. This hidden/invisible interaction also flourished because the professional organizations follow what Noble and Pym (1970, p. 438) termed a “collegial pattern of authority” and stimulated an openness for trial and error. This could explain why some interactions within the industry remain hidden because there is careful selection of trusted collaborators who share a set of skills associated with a specific professional association.

### Recognizing and Configuring Emerging Forms of Coordination As Response to Global Plant Diseases

The case study of emerging forms of coordination in distinct organizational fields, i.e., industry-based and formalized partnerships, temporary and *ad hoc* multi-stakeholder settings, and professional associations and networks. These organizational fields were shaped by both formal organizational structures and informal preferences identified distinct forms of coordination. Tracing a variety of interaction processes and encounters in these organizational fields and matching these with theoretical accounts enabled us to typify emerging forms of coordination. Based on this typology, we propose a set of qualitative indicators to recognize these three forms, acknowledging that coordination is difficult to quantify (**Table 5**). These indicators based on our theory-informed typology may inform stakeholders involved in management other plant diseases in banana or other crops to recognize and assess the plausible emergence of forms of coordination. The three typical forms serve as a sufficiently exhaustive representation of coordination to guide an integrative plant disease management approach, while leaving space for additional forms of coordination.

**Table 5:** Indicators to recognize emerging forms of coordination.

Descriptions	Forms of coordinated action		
	Rule-based	Cognition-based	Skill-based
Action and events	Conduct mandated activities, formulate, and follow rules	Organize activities that generate or share information	Accomplish research, development, and extension activities with long standing connections/partners
Composition	Requires regular organizational membership	Coordinate interim/temporary, multi-disciplinary members	Established by specialists or professional groups
Organization set-up	Formal and structured	*Ad hoc* and flexible	Dynamic and cross organizational boundaries
Objectives	Advocacy, formulation, and imposition of policies	Knowledge generation and finding solutions/answers	Implementation and application of solutions
Orientation	Bureaucratic	Exchange of information	Problem solving
Specific type of response	Recommending policies, implementation of rules	Creating Task Forces and activities that require interactions and dialogues	Formulating and evaluating specific technical solutions

This paper considers the emergence and precise forms of coordination as highly contingent on both the characteristics of the disease itself, i.e., the long-term persistence in the soil and the uncertainties related to spread and control in the case of TR4, and the area-specific history of actor constellations, i.e., in the case of the area in Mindanao the entangled presence of export-oriented multinational companies, processes of agrarian reform, and decentralized government responsibilities. Our area-based perspective used for the case study indicates that these contextual conditions importantly determine whether and how distinct forms of coordination both emerge and combine in an area affected by Foc TR4. The configuration of distinct forms of coordination is an important determinant for the possible emergence of a so-called collaborative community. According to Adler et al. (2008, p. 366), participants in a collaborative community “coordinate their activities through a shared commitment to a set of ultimate goals.” These authors show that the structure of a collaborative community is characterized by the combination of more global and open ties and stronger local ties. Its values are based on trust, with emphasis on contribution, concern, honesty, collegiality, and value-rationality. However, according to Kolbjørnsrud (2016), collaborative communities have challenges relating to governance such as mutual monitoring, membership restrictions, values and rules, and incentives. Our analysis indicates that the emergence of Foc TR4 influenced organizational dynamics in the Philippine banana sector. This may encourage the multinational banana companies to become part of a locally embedded collaborative community, as is suggested by Faulconbridge (2010). However, it is still difficult to assess whether this increases the likelihood of the formation of such a collaborative community, in which different actors converge to address a common problem.

## Conclusion

This paper expounded that coordination is one of the responses of a sector confronted with an emerging global plant disease affecting an important export variety embedded in a landscape of diverse production systems of the same crop. The paper exposed that the spread and emergence of Foc TR4 either changed or induced coordination among stakeholders with different interests, e.g., large-scale export or diverse food provision, or from different domains, e.g., private and public. Coordinated action prior to the emergence of TR4 was modified or intensified. Based on qualitative analysis and theory matching, the paper distinguished three forms of coordinated action: rule-based, cognition-based, and skill-based. The qualitative analysis indicates that formalized partnership importantly continued its routinized forms of coordination but also opened space for new collaborations. Multi-stakeholder setting offered opportunities for sense-making jointly with surfacing tensions and different perspectives on how to handle the unknowns and uncertainties attached to Foc TR4. Finally, it highlights the informal form of skill-based coordination as an important resource for navigating the uncertainties and tensions and engage with trusted collaborators in trial and error based experimentation and trials, which entail an unprecedented crossing of organizational boundaries. Our analysis suggests that integrative capacity to manage a global plant disease and its spread entails a combination of technical measures and conditions conducive to interactions and coordination among organizations and actors with different resources and diverse interests. We consider it important to recognize these different forms of coordination and understand how they combine in the specific regional circumstances of doing business and interactions between private and public sectors. A careful suggestion from this study is that, rather than in the form of formalized partnerships, a collaborative community may emerge from the invisible layer of professionals working in different organizations and crossing sectoral boundaries.

## Author Contributions

MM and SV contributed to research design, conceptualization, analysis and writing. LD contributed to analysis, writing and subsequent discussions.

## Funding

Interdisciplinary Research Fund of the Wageningen University and Research

## Conflict of Interest Statement

The authors declare that the research was conducted in the absence of any commercial or financial relationships that could be construed as a potential conflict of interest.
